# Multi-Omics Integrative Analysis Identifies the NK Cell–STAT3 Axis as a Shared Immunogenetic Hub Underlying the Comorbidity of Primary Sclerosing Cholangitis and Ulcerative Colitis

**DOI:** 10.3390/life16060950

**Published:** 2026-06-04

**Authors:** Ruiqi Zhao, Mengyao Han, Bei Zhang, Mengqing Ma, Kongli Fan, Jing Li, Jialing Sun, Xiaozhou Zhou

**Affiliations:** 1 The Fourth Clinical Medical College, Guangzhou University of Chinese Medicine, Shenzhen 518033, China; 20252120304@stu.gzucm.edu.cn (R.Z.);; 2 The Second Clinical Medical College, Guangzhou University of Chinese Medicine, Guangzhou 510006, China; 3 Faculty of Chinese Medicine, Macau University of Science and Technology, Taipa, Macao 999078, China

**Keywords:** primary sclerosing cholangitis, ulcerative colitis, genome-wide association study, single-cell transcriptomics, NK cells, *STAT3*, immune regulation

## Abstract

Primary sclerosing cholangitis (PSC) and ulcerative colitis (UC) exhibit a striking clinical comorbidity, with 60–80% of PSC patients concurrently harboring UC, yet the shared immunogenetic mechanisms remain poorly understood. Here, we constructed a multi-omics integrative framework to systematically dissect the cellular and molecular basis of this comorbidity. GWAS meta-analyses were performed for each disease, followed by tissue-level enrichment assessment using QTLEnrich, MAGMA, and gsMap spatial mapping. Single-cell transcriptomic atlases were constructed, and cell-type prioritization was conducted using four complementary methods. Core genes were identified through cross-validation of five algorithms, with subsequent genomic fine-mapping via FUMA and GCTA-COJO. Tissue-level analyses consistently identified the intestine and immune-related tissues as commonly affected. Multi-dimensional evidence integration prioritized natural killer (NK) cells as the core effector cell type for both diseases, supported principally by CELLECT (Cell-type Expression-specific Integration for Complex Traits) heritability enrichment and single-cell differential analysis. Convergence of five gene-level algorithms pinpointed *STAT3* as the sole high-confidence comorbidity gene, broadly expressed across immune cell populations and exhibiting tissue-differential alternative splicing. Colocalization identified a high-risk variant (rs3736161) within the *STAT3* locus, with conditional analysis revealing 35 additional independent signals. These findings identify the NK cell–*STAT3* axis as a central immunogenetic hub connecting PSC and UC, offering potential therapeutic targets for comorbidity management.

## 1. Introduction

Primary sclerosing cholangitis (PSC) is a chronic cholestatic liver disease characterized by progressive inflammation and fibrosis of the intrahepatic and extrahepatic bile ducts, ultimately leading to biliary strictures, cirrhosis, and hepatic failure [1]. No pharmacological therapy has been validated through randomized controlled trials, and liver transplantation remains the only curative option for patients with end-stage disease [1]. Ulcerative colitis (UC) is a chronic, non-specific inflammatory bowel disease affecting the colonic and rectal mucosa, manifesting primarily as recurrent bloody diarrhea, abdominal pain, and mucoid stool, with a steadily increasing global incidence [2].

Notably, PSC and UC share a remarkably high clinical comorbidity: approximately 60–80% of PSC patients have concomitant UC, and the prevalence of PSC among UC patients is substantially higher than that in the general population [1,3]. This pronounced co-occurrence strongly implies that the two diseases may share underlying genetic susceptibility and immunopathological mechanisms, yet the molecular basis driving this comorbid association has not been fully elucidated.

From an immunological standpoint, both PSC and UC are considered immune-mediated diseases. In PSC, aberrant immune cell infiltration into the periductal region and the consequent release of pro-inflammatory cytokines lead to biliary epithelial injury and progressive fibrosis [1]. In UC, dysregulation of both innate and adaptive mucosal immunity sustains a chronic inflammatory response, with aberrant activation of cytokine networks regarded as a core pathological driver [2,4]. The “gut–liver axis” hypothesis posits that gut-derived immune cells and inflammatory mediators can reach the liver via the portal venous circulation, triggering bile duct injury and thereby serving as a critical link between the two diseases [5]. However, the specific immune cell subsets and core regulatory molecules that bridge the gut–liver immune axis remain to be systematically defined at the genetic level.

Genome-wide association studies (GWAS) have provided important clues regarding the genetic underpinnings of PSC and UC. Previous GWAS have identified multiple risk loci for each disease, some of which show genetic overlap between the two conditions [6]. Cross-trait genetic analyses of multiple immune-mediated diseases have further uncovered a shared genetic architecture across autoimmune disorders [7]. Nevertheless, conventional GWAS have primarily focused on individual disease signal discovery and lack in-depth, systematic dissection of the shared mechanisms underlying PSC–UC comorbidity. Moreover, the majority of GWAS-identified risk loci reside in non-coding genomic regions, making it difficult to directly infer causal genes and effector cell types [6].

In recent years, the development of multiple post-GWAS analytical approaches has provided powerful tools to bridge this gap. Colocalization analyses of expression quantitative trait loci (eQTLs) and splicing QTLs (sQTLs), leveraging large-scale functional genomics resources such as GTEx [8], can link GWAS signals to gene expression regulation. Transcriptome-wide association studies (TWAS) aggregate SNP-level signals to the gene level through gene expression prediction models [9]. In particular, cell-type prioritization algorithms [10] have enabled the resolution of GWAS signals at the cell-type level. Concurrently, advances in single-cell RNA sequencing (scRNA-seq) technology have provided unprecedented resolution for directly observing disease-state changes in cellular composition and transcriptional programs [10].

Despite these advances, no study has yet systematically integrated multi-layered omics data to comprehensively dissect the shared immunogenetic mechanisms of PSC–UC comorbidity across tissue, cellular, and gene levels. Accordingly, the present study aimed to construct a multi-dimensional integrative analytical framework: at the tissue level, to identify commonly affected tissues through QTLEnrich (v2) [8], MAGMA [11], and gsMap (v1.73.7) [12] spatial mapping; at the cellular level, to systematically prioritize key pathogenic cell types by integrating single-cell transcriptomic differential analysis with multiple GWAS-informed algorithms; at the gene level, to pinpoint core pathogenic genes through cross-validation using colocalization analysis, chromatin accessibility analysis, co-expression network analysis, and cell-type-specific TWAS; and at the variant level, to perform fine-resolution dissection through risk locus annotation and conditional analysis. Through this strategy, our study seeks to uncover the core immune cell types and key regulatory genes underlying PSC–UC comorbidity, to provide novel genetic evidence for understanding the molecular basis of the gut–liver immune axis, and to lay a theoretical foundation for the development of comorbidity-targeted therapeutic strategies.

## 2. Materials and Methods

### 2.1. Sources of Genome-Wide Summary Statistics

Our study focused on GWAS data for PSC and UC, each incorporating summary statistics from two independent studies. For PSC, GWAS data were obtained from the FinnGen database (https://www.finngen.fi/en, accessed on 13 December 2025; ID: K11_CHOLANGI; 2317 cases and 437,418 controls) and the IEU OpenGWAS database (https://opengwas.io/, accessed on 16 December 2025; ID: ieu-a-1112; 2871 cases and 12,019 controls). For UC, GWAS data were similarly retrieved from the FinnGen database (ID: K11_UC_STRICT2; 7220 cases and 492,160 controls) and the IEU OpenGWAS database (ID: ukb-b-19386; 1987 cases and 461,023 controls).

### 2.2. Single-Cell Multiome (ATAC + Gene Expression) Dataset from Healthy Donor PBMCs

The peripheral blood mononuclear cell (PBMC) dataset from healthy donors was obtained from a publicly accessible dataset on the 10× Genomics platform (https://www.10xgenomics.com/datasets/pbmc-from-a-healthy-donor-granulocytes-removed-through-cell-sorting-10-k-1-standard-1-0-0; single-cell multi-omics data from healthy human PBMCs, accessed on 16 December 2025) and is distributed under the Creative Commons Attribution 4.0 International (CC BY 4.0) license. The dataset contains a median of 1826 genes and 3776 unique molecular identifiers (UMIs) per cell for gene expression, and 13,486 high-quality fragments per cell for chromatin accessibility, covering 108,377 open-chromatin peaks and 15,494 genes, with 85,468 peak–gene links identified. Further details are provided in the Data Availability Statement.

### 2.3. Single-Cell Transcriptomic Data

Single-cell transcriptomic data for PSC were obtained from the publicly available Gene Expression Omnibus (GEO) dataset GSE247128, from which 8 PSC samples and 6 healthy control samples were selected for downstream analysis. Single-cell transcriptomic data for UC were obtained from GEO dataset GSE250487, comprising 8 UC samples and 4 healthy controls.

### 2.4. Quality Control

A series of quality control (QC) procedures were applied to the GWAS data prior to analysis to ensure data accuracy and reliability. The minor allele frequency (MAF) was calculated for each SNP, and variants with MAF < 0.01 were excluded to reduce the uncertainty associated with low-frequency variants. All GWAS data were converted to the GRCh38/hg38 genome build to ensure format consistency for downstream analyses. Given the strong association of the major histocompatibility complex (MHC) region on chromosome 6 with autoimmunity and its extensive linkage disequilibrium (LD), which could confound GWAS analyses, SNPs located within the MHC region were excluded.

For single-cell transcriptomic data, a systematic QC pipeline was implemented. Data were loaded and initialized using the Seurat package (version 5.0.0) [13], and the proportions of mitochondrial genes and hemoglobin genes (including HBA1, HBA2, and HBB) were calculated to assess cell quality. Stringent filtering criteria were applied to retain high-quality cells: a minimum of 1000 unique molecular identifiers (UMIs) per cell, between 200 and 5000 detected genes per cell, mitochondrial gene proportion ≤ 15%, and hemoglobin gene proportion ≤ 3%. Dimensionality reduction was performed using principal component analysis (PCA) followed by Uniform Manifold Approximation and Projection (UMAP). The Harmony algorithm [14] was applied to correct for batch effects arising from sample origin.

### 2.5. Genome-Wide Meta-Analysis

To enhance statistical power, a fixed-effect model GWAS meta-analysis was performed by separately integrating GWAS summary statistics from two independent cohorts for each trait, thereby improving the ability to detect small-effect genetic variants.

### 2.6. Tissue-Specific eQTL/sQTL Enrichment Analysis Using QTLEnrich

Expression quantitative trait loci (eQTLs) and splicing quantitative trait loci (sQTLs) across 49 tissues from GTEx v8 were used to evaluate tissue-level relevance for PSC and UC. QTLEnrich is a rank-based and permutation-based method that assesses whether phenotypic associations are enriched among eQTLs and sQTLs in specific tissues and quantifies the statistical significance of such enrichment. The method accounts for three potential confounders: minor allele frequency (MAF), distance to the transcription start site (TSS) of the target gene, and local linkage disequilibrium (LD). Adjusted fold enrichment and enrichment *p*-values were used to evaluate the significance of QTLEnrich results.

### 2.7. Tissue-Specific MAGMA Enrichment Analysis

To complement the QTLEnrich analysis, MAGMA enrichment analysis was performed to explore the tissue-specific genomic features associated with PSC and UC. GWAS summary data for both traits were formatted for MAGMA input, and gene-level enrichment analyses were conducted. Tissues with *p* < 0.01 were considered credibly enriched.

### 2.8. Spatial Transcriptomic Mapping of Tissue Enrichment Specificity for PSC and UC

To investigate the spatial distribution of PSC- and UC-associated cellular signals, we integrated single-cell spatial transcriptomic (sc-ST) data with GWAS summary statistics using the genetically informed spatial mapping of cells for complex traits (gsMap) algorithm [12]. This algorithm integrates cross-species analyses spanning mouse embryonic and brain tissues, macaque cerebral cortex, and human GWAS data to identify the spatial distribution patterns of disease-associated cell populations. Its core principle involves mapping GWAS-derived trait-associated gene expression patterns onto spatially resolved cells to evaluate the association between specific anatomical regions and complex traits at cellular resolution. Based on spatial transcriptomic atlases of E16.5 mouse embryonic tissues (covering 25 organs), we generated PSC- and UC-specific enrichment maps and spatial gene expression profiles, establishing phenotypic spatial pathogenesis maps at single-cell resolution.

### 2.9. Biological Pathway Enrichment Analysis Using GeneEnrich

A gene set enrichment analysis approach was adopted to systematically analyze the genetic variants and expression profiles associated with PSC and UC. Using the GeneEnrich tool, hypergeometric and permutation tests were performed to evaluate the enrichment of candidate gene sets in biologically relevant functional or phenotypic gene sets. Empirical *p*-values were calculated via permutation testing to mitigate tissue-specific bias. Functional gene sets were sourced from Gene Ontology (GO), Reactome, the Kyoto Encyclopedia of Genes and Genomes (KEGG), the Molecular Signatures Database (MSigDB), and Mouse Genome Informatics (MGI). An empirical *p* < 0.05 within each database was considered nominally significant.

### 2.10. Single-Cell Transcriptomic Atlas Annotation for Identification of Cellular Signals in PSC and UC

Preprocessed single-cell data were subjected to multi-dimensional visualization using Harmony-corrected dimensionality reduction results. Cell neighborhood graphs were constructed based on Harmony-reduced embeddings, and a multi-resolution clustering strategy (32 resolutions ranging from 0.01 to 3.0) was employed. The optimal resolution was determined via clustering tree evaluation, yielding stable cell subpopulations. To characterize the molecular features of each cluster, differentially expressed marker genes were identified using the FindAllMarkers function (minimum expression fraction of 25%; log fold-change threshold of 0.25), and heatmaps were generated based on these marker genes. Cell-type annotation was performed automatically using the SingleR algorithm [15]. To assess whether cell-type proportions differed significantly between disease and control groups, the relative abundance of each cell type was calculated for each individual sample as the number of cells of that type divided by the total number of cells in that sample. The resulting per-sample proportions were then compared between groups using the Wilcoxon rank-sum test, with each donor sample treated as the independent unit of observation.

### 2.11. Identification of PSC- and UC-Associated Cellular Signals Using the seismicGWAS Method

seismicGWAS [16] introduces the Seismic framework, which computes a novel cell-type specificity score that captures both the expression intensity and consistency across different cell types. Compared with methods such as scDRS, FUMA, and S-MAGMA for integrating GWAS and single-cell data, seismicGWAS robustly and efficiently detects cell-type–trait associations by incorporating a specificity score that accounts for gene expression variability, thereby avoiding arbitrary threshold selection. Specifically, GWAS summary statistics containing MAGMA Z-scores and cell-type-annotated single-cell transcriptomic data were imported, and the seismicGWAS R package was used to compute a gene-level specificity score for each cell type. This score reflects two aspects: (i) whether the gene is expressed at a consistently higher level in the target cell type compared with other cell types and (ii) whether the gene is expressed across all cells within that cell type. Cell-type–trait associations were then calculated using specificity scores and MAGMA Z-scores. Cell types with *p* ≤ 0.05 were considered significantly associated with PSC or UC.

### 2.12. Identification of PSC- and UC-Associated Cellular Signals Using the ECLIPSER Method

ECLIPSER [17,18] employs a Bayesian Fisher’s exact test against background GWAS locus scores to estimate cell-type-specific enrichment fold changes and *p*-values for each trait (GWAS locus set), tissue, and cell-type combination, with the cell-type specificity threshold set at the 95th percentile of background locus scores. The Bayesian approach estimates 95% confidence intervals for the enrichment fold change and is applicable to traits with few loci or where no locus exceeds the enrichment threshold. The analysis utilizes multi-level annotation data obtained from functional genomics platforms. Briefly, significant genetic loci associated with the target trait were first identified from GWAS results and then expanded using LD proxy relationships (r^2^ > 0.8) to comprehensively capture potential functional variants. During the preparatory stage, Wilcoxon rank-sum tests were performed for between-group differential expression analysis within each cell type, with a minimum of 3 cells per group, a minimum gene expression fraction of 10%, and a log_2_FC threshold of 0.5. Genes meeting the differential expression criteria (adjusted *p* < 0.05 and |log_2_FC| > 0.5) were retained. Cell types with *p* ≤ 0.05 were deemed to exhibit statistically significant enrichment.

### 2.13. CELLECT Analysis for Identification of PSC- and UC-Associated Cellular Signals

To evaluate the contribution of cell-type-specific gene expression to disease heritability, we employed two complementary methods within the CELLECT (Cell-type Expression-specific Integration for Complex Traits) framework: heritability-based stratified LD score regression (S-LDSC) [19] and gene-set-based MAGMA gene analysis [11]. S-LDSC was applied to the PSC and UC GWAS summary statistics. The baseline cell-type dataset was derived from the Tabula Muris reference [20]. LD scores were calculated using European samples from the 1000 Genomes Project Phase 3 as a reference panel. *p* < 0.05 was considered indicative of significant enrichment. The MAGMA gene set analysis module tested the correlation between gene-level association statistics and cell-type-specific mean gene expression levels. MAGMA performed pairwise conditional analyses for cell-type combinations to identify cell types whose association signals were independent of other prominent cell types. As with S-LDSC, a significance threshold of *p* < 0.05 was applied.

### 2.14. Multi-Dimensional Evidence Integration for Cell-Type Prioritization

To prioritize the most likely pathogenic cell types for PSC and UC, we consolidated evidence from four analytical approaches: (i) single-cell atlas annotation and differential analysis, (ii) ECLIPSER analysis, (iii) seismicGWAS analysis, and (iv) CELLECT analysis. For simplicity, each line of evidence was assigned equal weight (1 point), and the total score for each cell type was calculated as the sum across all evidence streams. A higher total score indicated a greater likelihood of the cell type being a key participant in disease pathogenesis [21].

### 2.15. Weighted Gene Co-Expression Network Analysis of Prioritized Cell Types to Identify Core Module Genes

The high-dimensional Weighted Gene Co-expression Network Analysis (hdWGCNA) method [22] was applied to perform weighted gene co-expression network analysis on the cell type identified through multi-dimensional evidence integration. Annotated single-cell Seurat objects were loaded, and the target cell type was specified for analysis. The WGCNA function was used to initialize the analysis environment, selecting genes expressed in at least 5% of cells as the candidate gene set. Metacells (k = 25) were constructed using the MetacellsByGroups function based on cell type and sample origin to reduce single-cell data sparsity while preserving biological variability. Metacell expression data were then normalized and scaled. Following PCA-based dimensionality reduction, the Harmony algorithm was applied to correct for batch effects arising from sample origin. For network construction, the SetDatExpr function was used to extract the expression matrix of the target cell type, and the TestSoftPowers function was employed to test soft-thresholding powers. An appropriate soft threshold was selected to construct a signed topological overlap matrix (TOM) network. Co-expression modules were identified using a dynamic tree-cutting algorithm, and module eigengene values were calculated. Module–cell type associations were further analyzed.

### 2.16. eCAVIAR Colocalization Analysis for PSC and UC

To identify high-confidence genes and regulatory mechanisms (eQTLs/sQTLs) potentially mediating the association at common risk loci for PSC and UC, we employed the Bayesian colocalization method eCAVIAR, which assesses whether co-occurring GWAS and eQTL/sQTL signals tag the same causal variant or haplotype, thereby accounting for local LD and allelic heterogeneity [23]. eCAVIAR incorporates built-in fine-mapping functionality and can process large-scale GWAS summary statistics. A maximum of two independent causal variants per locus was assumed. Input data comprised Z-scores (effect size beta divided by standard error) for each variant from both the GWAS and GTEx eQTL/sQTL studies. The LD window around each lead variant was defined as the chromosomal region containing variants with r^2^ > 0.1 (calculated using the 1000 Genomes Project Phase 3 as a reference panel), extended by 50,000 bp on each side. A colocalization posterior probability (CLPP) exceeding 0.01 was considered significant.

### 2.17. fastENLOC Colocalization Analysis for PSC and UC

To further refine the identification of genes and regulatory mechanisms mediating the association at common risk loci, we employed the Bayesian colocalization method fastENLOC [24]. This method uses its embedded DAP-G algorithm to perform fine-mapping on both GWAS and eQTL/sQTL loci separately, estimating the posterior probability that each variant is causal and then evaluating the colocalization probability that the two signals share the same causal variant. Importantly, fastENLOC does not impose an upper limit on the number of independent causal variants per locus. Input data comprised Z-scores for each variant from the GWAS and GTEx eQTL/sQTL summary data. The LD window for each lead variant was defined as described above for eCAVIAR. Colocalization results were expressed as the regional colocalization probability (RCP). Following the method’s recommendations, an RCP exceeding 0.1 was considered evidence of significant colocalization.

### 2.18. Open Chromatin to Gene Expression Analysis

The Open4Gene analysis aimed to explore genes expressed in immune cells from normal tissues and to determine whether stably expressed genes in immune cells exhibit potential associations with specific cell types identified in the PSC and UC single-cell transcriptomic atlases. Open4Gene is a Hurdle model-based statistical method specifically designed to handle the zero-inflated characteristics common in single-cell data. The analytical pipeline consisted of several key steps: scRNA-seq and assay for transposase-accessible chromatin with sequencing (ATAC-seq) data were first normalized, dimensionally reduced, and clustered using the Seurat and Signac packages. RNA expression matrices, ATAC peak matrices, and cell metadata (including cell-type annotations and technical covariates) were extracted from the Seurat objects. ATAC peaks were then linked to gene promoter regions using a window size of 100 kb to define peak–gene pairs. A two-component Hurdle model was employed to test the association of each peak–gene pair: the zero component used a binomial distribution (logit link function) to model the relationship between ATAC open-chromatin status and the probability of zero gene expression; the count component used a truncated negative binomial distribution (log link function) to model the relationship between ATAC signal intensity and non-zero gene expression levels [25].

### 2.19. Identification of Candidate Genes Using scPrediXcan

scPrediXcan [26] is a cell-type-specific association analysis framework that integrates state-of-the-art deep learning methods with a conventional transcriptome-wide association study (TWAS) framework. The method predicts epigenomic features from DNA sequences, enabling high-precision prediction of cell-type-specific gene expression and capturing complex gene regulatory syntax overlooked by linear models. The scPrediXcan framework consists of three core steps: first, a ctPred model is constructed using a multi-layer perceptron (MLP) to predict gene expression percentiles based on epigenomic data and observed cell-type-specific gene expression. Second, the deep learning model is linearized into an SNP-based elastic net model (ℓ-ctPred) compatible with GWAS summary statistic-based association testing. Finally, ℓ-ctPred and the S-PrediXcan framework are used to execute TWAS at the cell-type level, testing gene–trait associations across different cell types.

### 2.20. Genomic Risk Locus Analysis for PSC and UC

Genomic risk locus identification and annotation were performed using the FUMA platform (https://fuma.ctglab.nl/snp2gene, accessed on 18 February 2026). GWAS summary files for PSC and UC, containing SNP identifiers and LD reference information, were uploaded. The FUMA platform performed initial quality control to remove missing values and low-quality SNPs. A genome-wide significance threshold of *p* < 5 × 10^−8^ was applied to identify risk loci associated with PSC and UC.

### 2.21. Conditional Analysis of Genomic Risk Loci

Conditional analysis was performed on the high-confidence risk loci identified for PSC and UC to investigate whether independent secondary association signals existed within the significantly colocalized GWAS signal regions. Specifically, the lead variant at each locus was used as a conditioning variable, and conditional association analysis was performed on the GWAS summary statistics using the COJO tool in the GCTA software (v1.94.1) [27]. To ensure inclusion of low-frequency lead variants, variants with MAF < 0.0001 were pre-filtered. Variant allele frequencies required for the analysis were obtained from the 1000 Genomes Project.

### 2.22. Prioritized Gene Expression Annotation and Exon-Level Expression Analysis

Exon-level expression data from the GTEx portal (https://gtexportal.org/, accessed on 22 February 2026) were used to annotate prioritized genes, enabling analysis of transcript isoform diversity, structural features of each transcript, and tissue-specific expression distributions across human tissues.

## 3. Results

### 3.1. Genome-Wide Meta-Analysis of PSC and UC

Through separate meta-analyses of GWAS summary statistics, we successfully enhanced the statistical power of two independent cohorts to detect genetic variants associated with PSC and UC. Following rigorous quality control, a total of 9,843,954 genome-wide SNPs were retained for PSC and 9,255,516 for UC.

### 3.2. Enrichment of PSC and UC Associations in eQTLs and sQTLs

**Figure 1:**
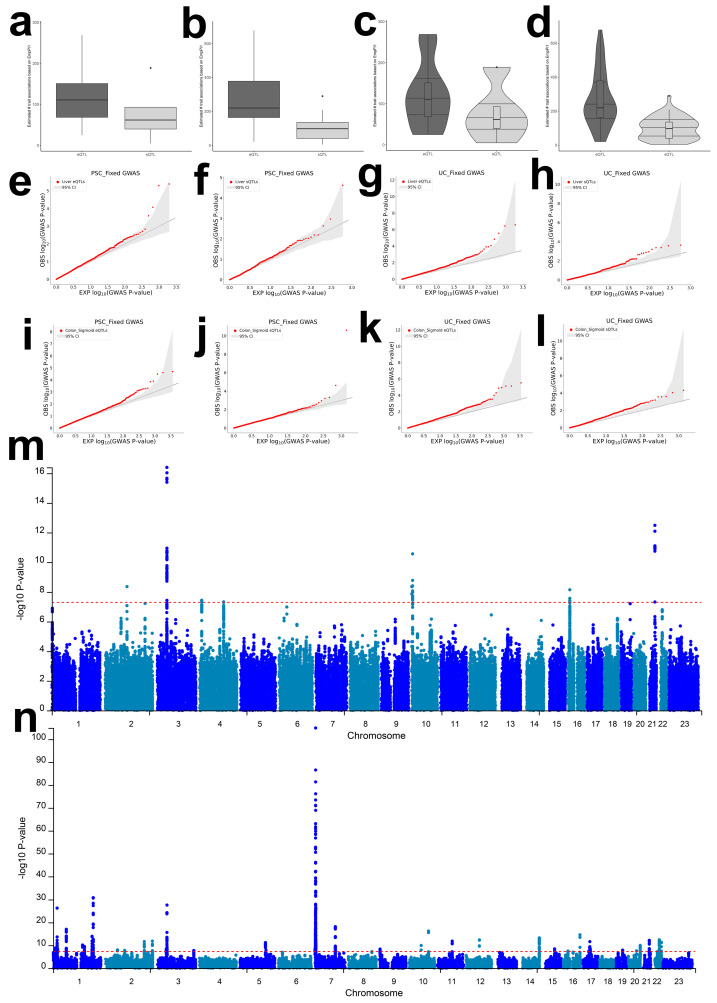
QTL enrichment analysis and genome-wide association landscapes for PSC and UC. (**a**,**b**) Box plots depicting the distribution of QTLEnrich statistics for (**a**) PSC and (**b**) UC. Dark gray denotes expression quantitative trait loci (eQTLs) and light gray denotes splicing quantitative trait loci (sQTLs). (**c**,**d**) Violin plots showing the distribution of QTLEnrich statistics for (**c**) PSC and (**d**) UC. Dark gray denotes eQTLs and light gray denotes sQTLs. (**e**) Quantile–quantile (QQ) plot of SNP-level *p* values for eQTLs in liver tissue from the PSC GWAS dataset. The x-axis represents the expected distribution of *p* values under the null hypothesis, and the y-axis represents the observed distribution. The gray shaded area indicates the expected null distribution with its 95% confidence interval. (**f**) QQ plot of SNP-level *p* values for sQTLs in liver tissue from the PSC GWAS dataset; axes and shading are as described in (**e**). (**g**) QQ plot of SNP-level *p* values for eQTLs in liver tissue from the UC GWAS dataset; axes and shading are as described in (**e**). (**h**) QQ plot of SNP-level *p* values for sQTLs in liver tissue from the UC GWAS dataset; axes and shading are as described in (**e**). (**i**) QQ plot of SNP-level *p* values for eQTLs in colon tissue from the PSC GWAS dataset; axes and shading are as described in (**e**). (**j**) QQ plot of SNP-level *p* values for sQTLs in colon tissue from the PSC GWAS dataset; axes and shading are as described in (**e**). (**k**) QQ plot of SNP-level *p* values for eQTLs in colon tissue from the UC GWAS dataset; axes and shading are as described in (**e**). (**l**) QQ plot of SNP-level *p* values for sQTLs in colon tissue from the UC GWAS dataset; axes and shading are as described in (**e**). (**m**) Manhattan plot of the PSC GWAS data. The red dashed line indicates the genome-wide significance threshold (*p* = 5 × 10^−8^). (**n**) Manhattan plot of the UC GWAS data. The red dashed line indicates the genome-wide significance threshold (*p* = 5 × 10^−8^).

To evaluate the potential organ-level associations of eQTLs and sQTLs with PSC and UC, we applied the QTLEnrich method. The significance and reliability of the eQTL/sQTL enrichment results were validated using box plots, violin plots, and quantile–quantile (QQ) plots, all of which demonstrated the robustness of the data included in this study (Figure 1a–l).

Manhattan plots for PSC (Figure 1m) and UC (Figure 1n) revealed significant peaks across multiple chromosomes. We then systematically tested whether eQTLs and sQTLs across 49 GTEx (v8) tissues were enriched within the association signals for PSC and UC. The results showed significant enrichment of both eQTLs and sQTLs for the genetic associations of PSC and UC across multiple GTEx tissues (see Supplementary Table S1 for detailed enrichment data). Taken together, the adjusted fold enrichment and estimated number of trait associations with regulatory effects indicated that the intestine is one of the key tissues cumulatively affected in both PSC and UC.

### 3.3. MAGMA Enrichment Analysis and Spatial Mapping Reveal the Tissue-Specific Basis of PSC and UC

**Figure 2:**
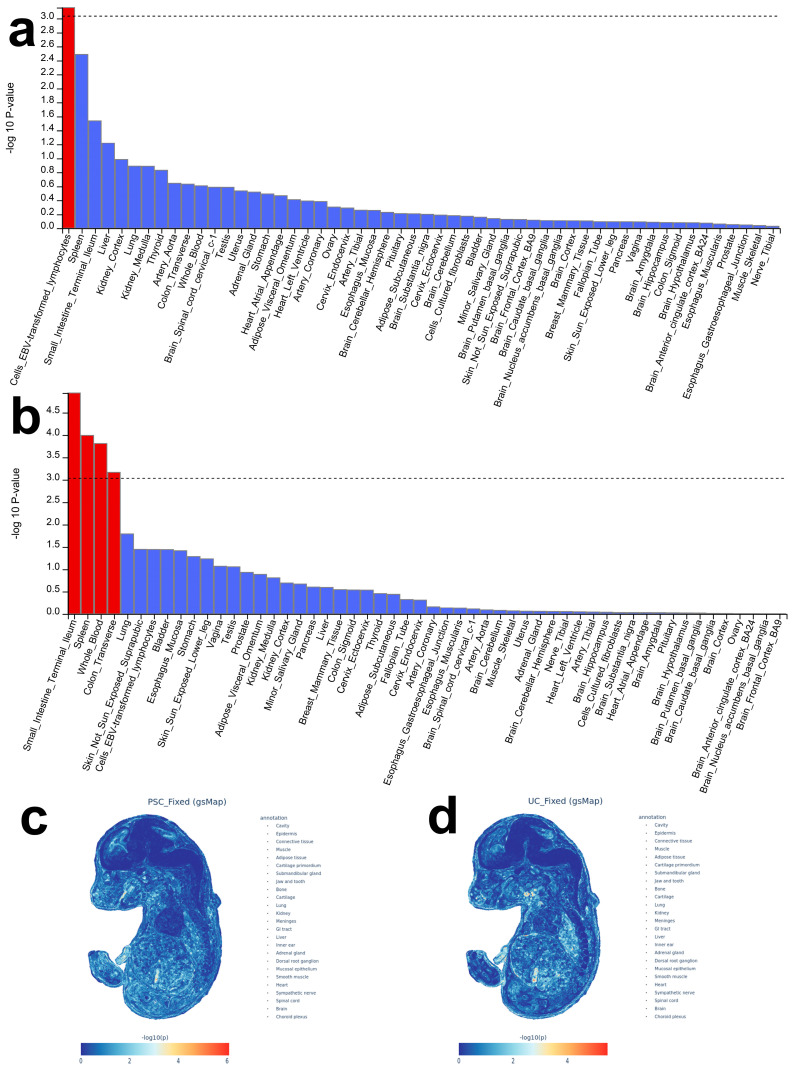
Tissue-level enrichment and spatial mapping of genetic signals for PSC and UC. (**a**,**b**) Bar plots showing tissue enrichment results from MAGMA analysis for (**a**) PSC and (**b**) UC. The x-axis represents different tissues, and the y-axis represents −log_10_(*p* value). The dashed line indicates the significance threshold at *p* = 0.001. (**c**) Spatial distribution map of PSC-associated genetic signals projected onto E16.5 mouse embryo data using the gsMap algorithm. Redder points indicate smaller *p* values. (**d**) Spatial distribution map of UC-associated genetic signals projected onto E16.5 mouse embryo data using the gsMap algorithm. Redder points indicate smaller *p* values.

To further explore the genetic basis of PSC and UC from a tissue-specific perspective, we performed MAGMA gene set enrichment analysis. The results revealed highly significant enrichment of PSC genetic signals in EBV-transformed lymphocytes (*p* < 0.05) (Figure 2a; Supplementary Table S2). UC genetic signals were significantly enriched in the small intestine terminal ileum, spleen, and whole blood (*p* < 0.001) (Figure 2b; Supplementary Table S2). gsMap successfully mapped the GWAS genetic signals of PSC and UC onto the spatial transcriptomic atlas of E16.5 mouse embryonic tissues, and significant enrichment was confirmed in the gastrointestinal tract (PSC, GI tract *p* = 0.027; UC, GI tract *p* = 0.019) (Figure 2c,d). These results suggest that the intestine may be the primary affected organ in both PSC and UC, highlight potential spatial pathways of action, and provide a tissue-level spatial perspective for understanding the pathological mechanisms of these diseases.

### 3.4. Gene Functional Enrichment Analysis Reveals Tissue-Specific Biological Processes

**Figure 3:**
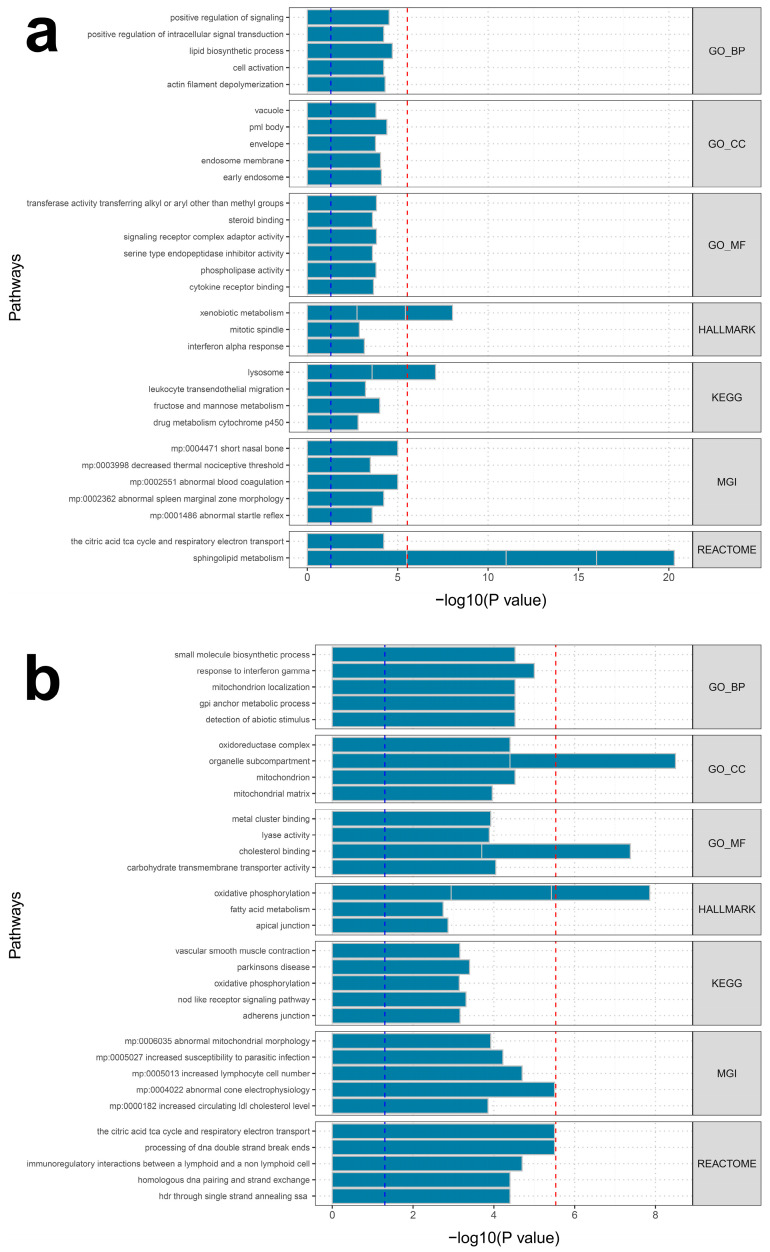
Pathway enrichment analysis of disease-associated eGenes and sGenes. (**a**) Pathway enrichment results of eGenes and sGenes significantly associated with PSC across multiple gene sets. (**b**) Pathway enrichment results of eGenes and sGenes significantly associated with UC across multiple gene sets. The x-axis represents −log_10_(*p* value). The blue dashed line indicates *p* = 0.05 and the red dashed line indicates *p* = 1 × 10^−5^. The y-axis represents the corresponding pathways.

To dissect the biological functions implicated by the genetic signals associated with PSC and UC, we used the GeneEnrich tool to systematically evaluate the functional enrichment of trait-associated genes in specific domains (see Supplementary Table S3 for detailed results). Genes significantly associated with both PSC and UC exhibited significant enrichment across multiple gene sets (Figure 3a,b). Specifically, significantly enriched biological processes for PSC included xenobiotic metabolism (HALLMARK; empirical *p* < 1 × 10^−5^), lysosome (KEGG; empirical *p* < 1 × 10^−5^), and sphingolipid metabolism (REACTOME; empirical *p* < 1 × 10^−5^). For UC, significantly enriched processes included organelle subcompartment (GO CC; empirical *p* < 1 × 10^−5^), cholesterol binding (GO MF; empirical *p* < 1 × 10^−5^), and oxidative phosphorylation (HALLMARK; empirical *p* < 1 × 10^−5^). These findings suggest that the genetic mechanisms of PSC and UC may operate through distinct biological pathways.

### 3.5. Cellular Composition of PSC and UC

**Figure 4:**
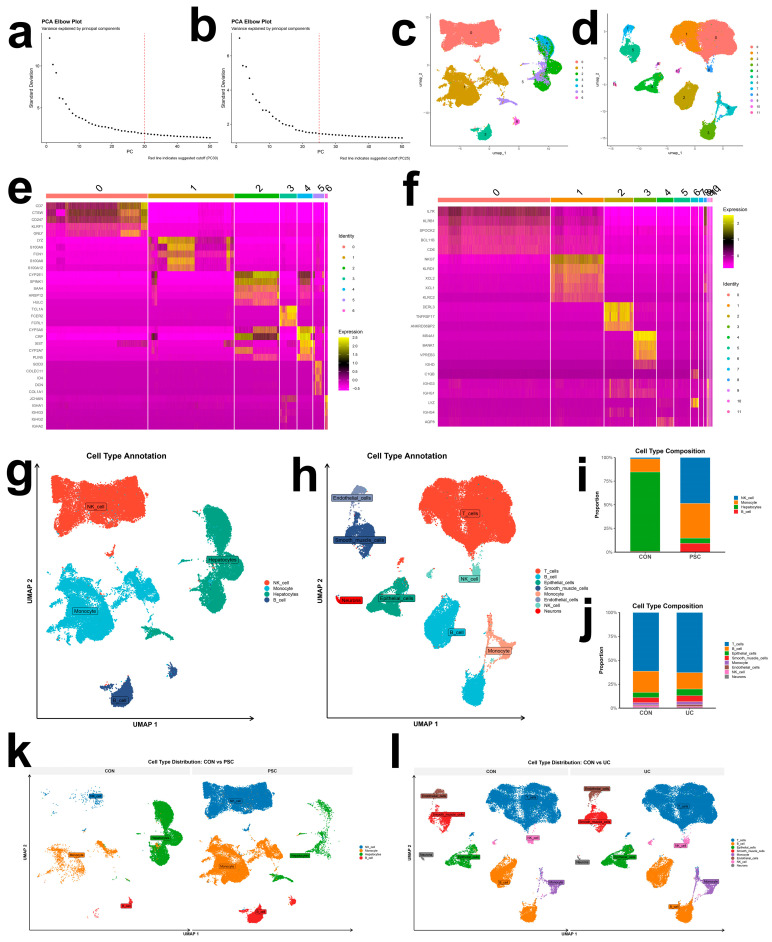
Single-cell transcriptomic characterization of PSC and UC. (**a**,**b**) Principal component analysis (PCA) elbow plots of single-cell transcriptomic data for (**a**) PSC and (**b**) UC. (**c**,**d**) Uniform Manifold Approximation and Projection (UMAP) visualization of single-cell transcriptomic data for (**c**) PSC and (**d**) UC. (**e**,**f**) Heatmaps of marker gene expression across distinct cell clusters identified in (**e**) PSC and (**f**) UC single-cell transcriptomic datasets. Yellow color intensity indicates higher expression levels. (**g**,**h**) UMAP plots following cell type annotation for (**g**) PSC and (**h**) UC single-cell transcriptomic data. (**i**,**j**) Cell type proportion distributions in (**i**) PSC and (**j**) UC single-cell transcriptomic datasets. “Con” denotes the control group. (**k**) UMAP plots showing the distribution of distinct cell types in control and PSC groups from PSC single-cell transcriptomic data. (**l**) UMAP plots showing the distribution of distinct cell types in control and UC groups from UC single-cell transcriptomic data.

In the single-cell transcriptomic atlas annotation phase, following rigorous data quality control, normalization, and Harmony correction in the low-dimensional embedding space, we performed systematic unsupervised clustering and cell-type identification on high-quality single-cell data. First, the quality of dimensionality reduction was assessed using elbow plots (Figure 4a,b).

Harmony-corrected reduced embeddings were then used to construct cell neighborhood graphs, and a multi-resolution clustering strategy was employed for community detection. Clustering tree analysis was performed to evaluate the stability of clustering results at different resolutions (Supplementary Figures S1 and S2), ultimately determining optimal resolutions of 0.01 for PSC and 0.1 for UC, which partitioned cells into distinct clusters (Figure 4c,d). Significantly upregulated marker genes were identified for each cluster and visualized in heatmaps (Figure 4e,f). Cell-type annotation was performed using the SingleR algorithm with reference datasets. In the PSC single-cell data, NK cells, monocytes, hepatocytes, and B cells were successfully annotated, while in the UC dataset, T cells, B cells, epithelial cells, smooth muscle cells, monocytes, endothelial cells, NK cells, and neurons were identified (Figure 4g,h). Cell-type proportions were visualized, revealing that NK cells constituted a notably higher proportion in the PSC group (Figure 4i), whereas cell-type proportions in the UC dataset appeared comparable between the UC and control groups (Figure 4j). Group-stratified UMAP plots were subsequently generated to more intuitively display the differences in cell-type proportions between disease and control groups (Figure 4k,l).

**Figure 5:**
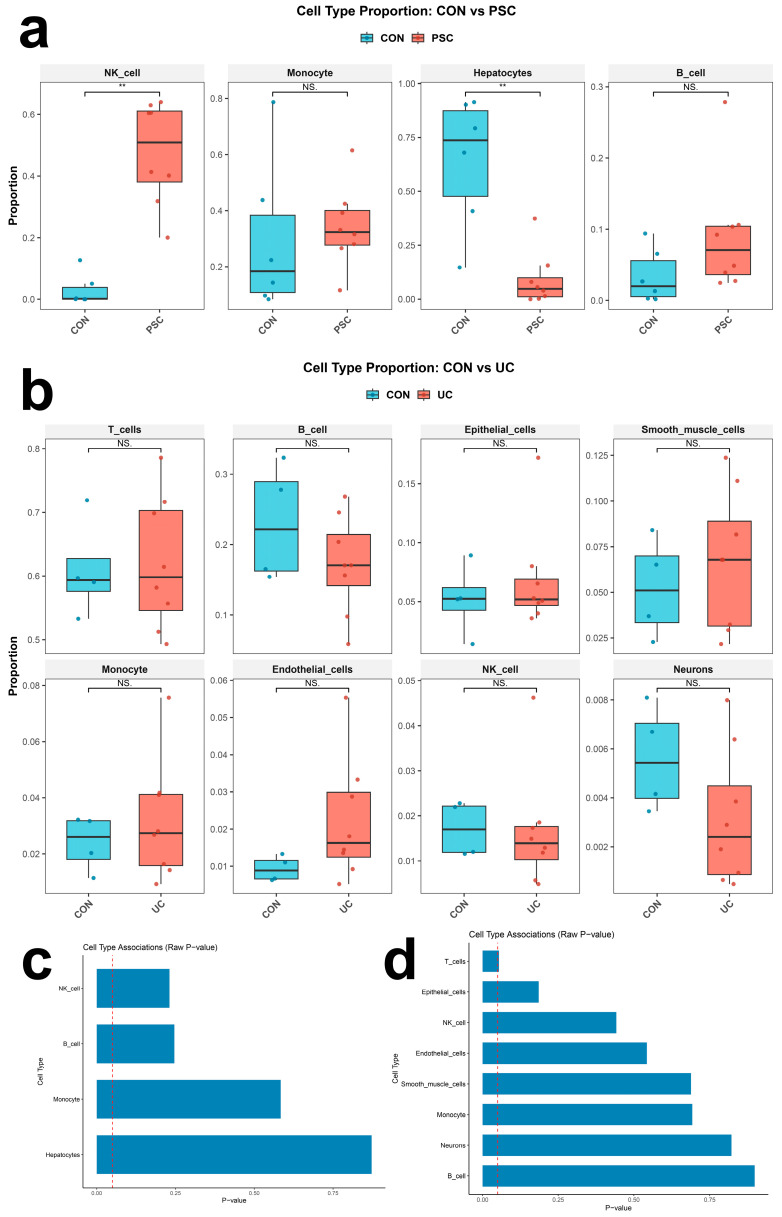
Cell type proportion alterations and TWAS-informed cell type prioritization in PSC and UC. (**a**,**b**) Box plots depicting cell type proportion differences in (**a**) PSC and (**b**) UC single-cell transcriptomic data. Blue represents the control group, and orange represents the disease group. Statistical significance was assessed using the Wilcoxon rank-sum test. NS., *p* > 0.05; **, *p* < 0.01. (**c**) Bar plot showing cell type prioritization results from seismicGWAS analysis of PSC single-cell transcriptomic data. The red dashed line indicates *p* = 0.05. (**d**) Bar plot showing cell type prioritization results from seismicGWAS analysis of UC single-cell transcriptomic data. The red dashed line indicates *p* = 0.05.

The Wilcoxon rank-sum test was then used to assess the statistical significance of cell-type proportion differences between disease and control groups (Figure 5a,b). NK cells and hepatocytes exhibited statistically significant differences between the PSC and control groups (*p* < 0.01), whereas no significant differences were observed for other cell types.

SeismicGWAS analysis revealed no cell type significantly associated with either PSC (Figure 5c) or UC (Figure 5d) (all *p* values > 0.05). Similarly, ECLIPSER cell-type-specific enrichment analysis showed no significant enrichment of genetic risk signals in any specific cell type for either trait (Table 1).

Using the CELLECT framework, S-LDSC analysis demonstrated significant enrichment of PSC heritability in hepatic natural killer cells (*p* < 0.05). UC heritability was likewise significantly enriched in hepatic natural killer cells (*p* < 0.05). MAGMA gene set analysis further validated and extended these findings, showing that NK cell-specific gene expression sets in the liver were significantly associated with both PSC and UC (*p* < 0.05) (see Supplementary Table S4 for detailed results).

### 3.6. Multi-Dimensional Evidence Integration for Cell-Type Prioritization

To systematically prioritize the most likely pathogenic cell types for PSC and UC, we integrated four complementary lines of computational biology evidence: (i) single-cell atlas annotation and differential analysis to identify cell types active in disease-relevant tissues; (ii) seismicGWAS and (iii) ECLIPSER analyses to evaluate GWAS signal enrichment in cell-type-specific regulatory elements based on functional genomics annotations; and (iv) CELLECT analysis (S-LDSC and MAGMA) to assess the contribution of cell-type-specific gene expression to disease heritability. The integrated analysis results (Table 2) clearly demonstrated that NK cells received the highest priority score, underscoring the central role of NK cells. Notably, these findings revealed the potential involvement of immune cells in the pathogenesis of PSC and UC.

### 3.7. Candidate Gene Networks

**Figure 6:**
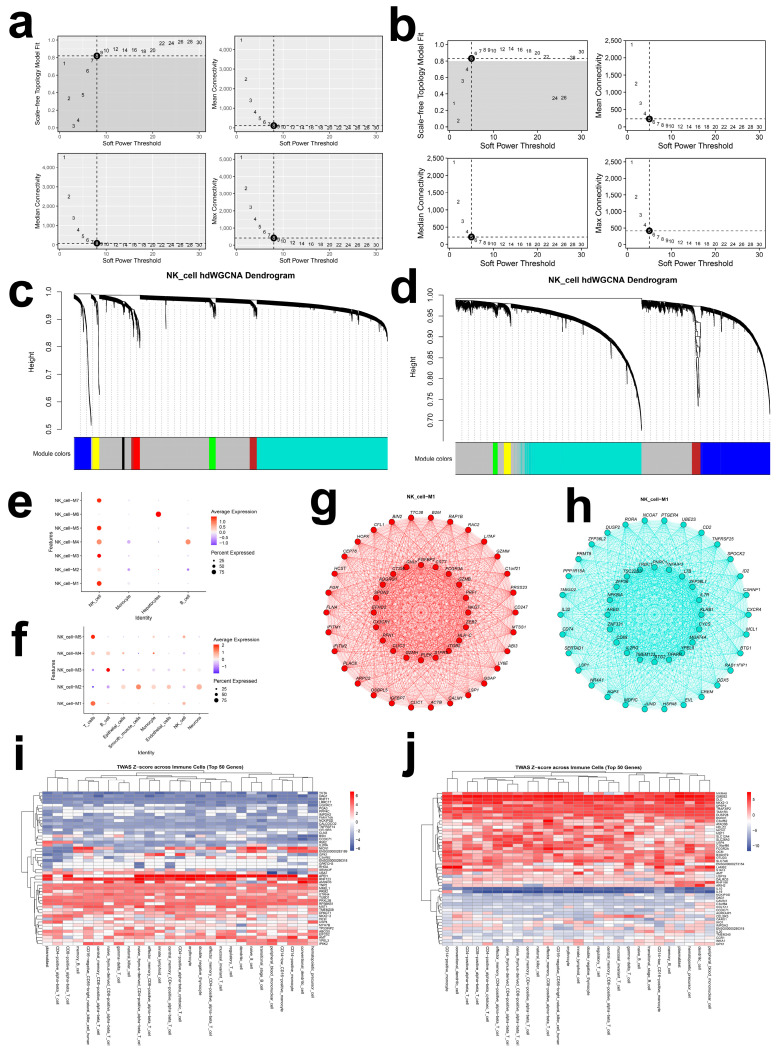
Co-expression network analysis and transcriptome-wide association with immune cell types. (**a**,**b**) Network topology plots displaying scale-free fit across a range of soft-thresholding powers from hdWGCNA analysis of (**a**) PSC and (**b**) UC single-cell transcriptomic data. (**c**) Co-expression network construction based on NK cells in PSC, showing the optimal soft threshold and dendrogram with module color assignments. (**d**) Co-expression network construction based on NK cells in UC, showing the optimal soft threshold and dendrogram with module color assignments. (**e**,**f**) Dot plots illustrating the expression profiles of distinct gene modules derived from NK cells across different cell types in (**e**) PSC and (**f**) UC single-cell transcriptomic data. (**g**) Gene network plot of the top 50 hub genes within the M1 module identified in NK cells from PSC single-cell transcriptomic data. (**h**) Gene network plot of the top 50 hub genes within the M1 module identified in NK cells from UC single-cell transcriptomic data. (**i**) Heatmap showing correlations between the top 50 genes identified by TWAS via scPrediXcan and 27 immune cell types for PSC. (**j**) Heatmap showing correlations between the top 50 genes identified by TWAS via scPrediXcan and 27 immune cell types for UC.

Based on the multi-dimensional evidence integration analysis described above, NK cells were identified as a potentially critical cell type involved in both PSC and UC. To dissect the transcriptional regulatory programs of NK cells in disease, we performed in-depth analysis using the hdWGCNA method. To overcome the inherent sparsity of single-cell data, metacells (k = 25) were constructed, and soft-thresholding powers of 8 (Figure 6a) and 5 (Figure 6b) were selected to build signed topological overlap matrix (TOM) networks, ensuring approximate scale-free topology. Using a dynamic tree-cutting algorithm, co-expression networks were successfully constructed and distinct modules identified (Figure 6c,d). Dot plots depicting the expression of different gene modules from NK cells across cell types showed that the M1 module exhibited consistently high expression in both the PSC (Figure 6e) and UC (Figure 6f) datasets. Network plots displayed the top 50 genes within the M1 module for PSC (Figure 6g) and UC (Figure 6h).

eCAVIAR analysis successfully identified multiple potentially causal genes and regulatory variants commonly associated with PSC and UC. A total of 7 loci (involving 65 genes) showed significant colocalization with PSC (CLPP > 0.01), and 37 loci (involving 217 genes) showed significant colocalization with UC (CLPP > 0.01).

Using the Bayesian colocalization method fastENLOC, systematic analysis of GWAS-significant loci against eQTL/sQTL signals across 49 GTEx (v8) tissues was performed. fastENLOC identified multiple genes with high colocalization probabilities. Seven loci (involving 75 genes) showed significant colocalization with PSC (RCP > 0.1) and 34 loci (involving 254 genes) with UC (RCP > 0.1). All colocalization results are provided in Supplementary Table S5.

Open4Gene Hurdle analysis identified 1455 significant cis-peak–gene regulatory pairs in NK cells (hurdle.Res.zero.*p* > 0.05, hurdle.Res.count.*p* < 0.05, and Spearman *p* < 0.05), involving 1064 genes (see Supplementary Table S6 for all results).

scPrediXcan was used to test gene–trait associations at the cell-type level (Supplementary Table S7, Figure 6i,j). In total, 1203 candidate genes significantly associated with PSC and UC were identified at the NK cell level.

### 3.8. Key Gene Cellular Expression Annotation and Exon-Level Splicing Event Analysis

**Figure 7:**
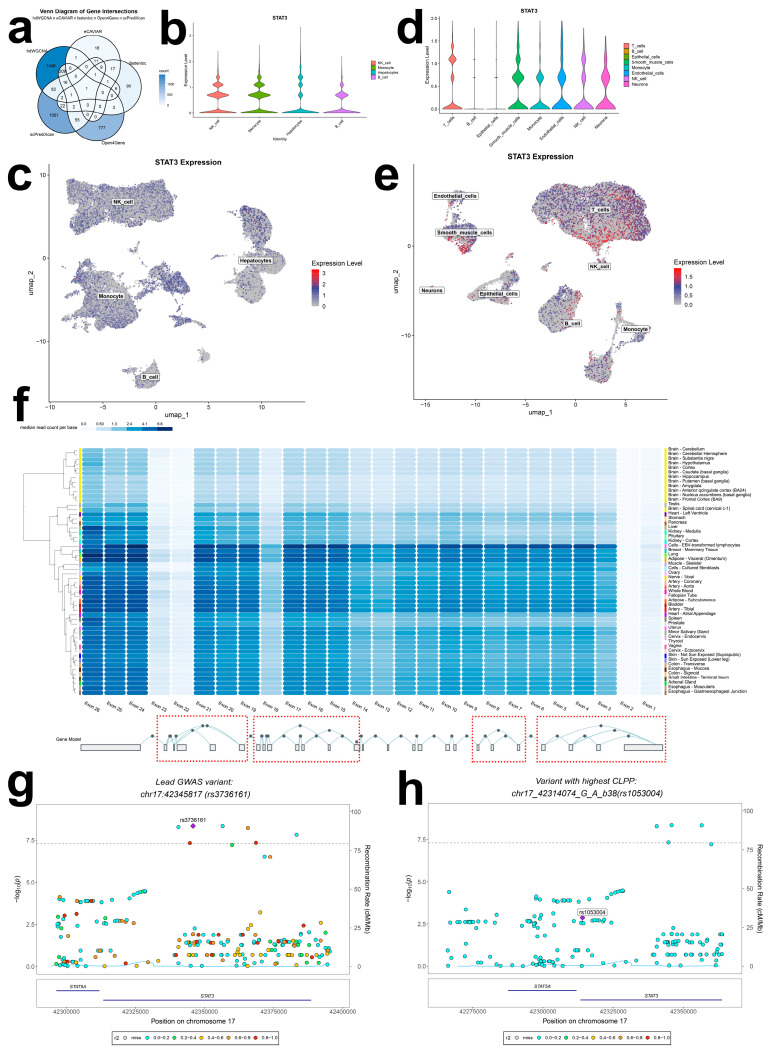
Identification and functional characterization of *STAT3* as a convergent key gene. (**a**) Venn diagram depicting the intersection of key genes identified by five complementary analytical approaches. (**b**) Violin plot showing *STAT3* expression levels across different cell types in PSC single-cell transcriptomic data. (**c**) UMAP plot of *STAT3* expression in PSC single-cell transcriptomic data; redder color indicates higher expression levels. (**d**) Violin plot showing *STAT3* expression levels across different cell types in UC single-cell transcriptomic data. (**e**) UMAP plot of *STAT3* expression in UC single-cell transcriptomic data; redder color indicates higher expression levels. (**f**) Exon cassette model of the *STAT3* gene. Connecting lines represent splicing events between all exons. The red dashed box highlights the putative alternative splicing site. The heatmap above displays tissue-level expression of the gene, with deeper blue indicating higher expression. The figure panel was adapted from the GTEx Portal (https://gtexportal.org/; accessed on 10 March 2026). (**g**) LocusZoom plot showing the colocalization locus of rs3736161 on chromosome 17. (**h**) LocusZoom plot showing the colocalization locus most strongly correlated with rs3736161 for rs1053004 on chromosome 17. Each dot represents an SNP, with redder color indicating a higher linkage disequilibrium (r^2^) value relative to the lead variant.

To further delineate the cellular origins of key pathogenic genes, we integrated multiple lines of gene prioritization evidence (Table 3) and obtained the intersection of the five algorithms described above (Figure 7a). A single gene, *STAT3*, was identified as the highest-confidence candidate. In the PSC single-cell transcriptomic data, both violin plots (Figure 7b) and UMAP plots (Figure 7c) demonstrated that *STAT3* was highly expressed across multiple cell types. In UC, *STAT3* was also broadly expressed in multiple cell types, including NK cells (Figure 7d,e). Exon-level analysis revealed that *STAT3* was expressed at particularly high levels in EBV-transformed lymphocytes, consistent with the tissue enrichment analysis results described above. Relatively high expression was also observed in colon and liver tissues, with lower expression in other tissues. This tissue-differential expression pattern is consistent with the possibility of alternative splicing events occurring in the Exon 1–4, 5–7, 14–19, and 21–25 regions, although direct isoform-level quantification would be required to formally establish this (Figure 7f).

### 3.9. Genomic Risk Loci

Based on the colocalization analyses described above [28], one high-risk genomic locus (rs3736161) was identified on chromosome 17, located within the *STAT3* gene (Figure 7g). Additionally, rs1053004 (Figure 7h) was identified as being in high LD with the lead SNP rs3736161. Subsequently, GCTA-COJO conditional analysis, conditioning on rs3736161, was performed to test the independence of 156 risk variants within the associated locus (Supplementary Table S8). Thirty-five variants, including rs1053004, remained significant after conditioning (*P*_conditional analysis < 0.05), indicating the existence of additional independent association signals beyond the lead SNP rs3736161. The genetic effects of these SNPs could not be explained by rs3736161, suggesting that multiple functional variants at this locus independently contribute to disease risk.

## 4. Discussion

By constructing a systematic multi-omics integrative analytical framework, this study provided an in-depth dissection of the shared immunogenetic mechanisms underlying PSC–UC comorbidity across tissue, cellular, and gene levels. The core findings can be summarized at three levels: (1) the intestine and immune-related tissues represent commonly enriched tissues for the genetic signals of both PSC and UC; (2) NK cells constitute the core immune effector cell type driving comorbidity between the two diseases; and (3) *STAT3* serves as a key hub gene connecting the immunopathological mechanisms of PSC and UC, with its locus harboring multiple independent functional variants.

### 4.1. The Intestine and Immune-Related Tissues: The Common Tissue-Level Foundation of PSC–UC Comorbidity

QTLEnrich analysis confirmed widespread tissue-specific enrichment of eQTLs and sQTLs among the GWAS association signals for both PSC and UC, with intestinal tissues showing significant enrichment for both diseases. MAGMA tissue enrichment analysis further revealed that PSC genetic signals were highly enriched in EBV-transformed lymphocytes, while UC signals were significantly enriched in the small intestine terminal ileum, spleen, and whole blood. These results collectively point to the immune and digestive systems as the shared genetic foundation of PSC and UC. The enrichment of PSC genetic signals in lymphocytes aligns with its nature as an immune-mediated disease [1], while the enrichment of UC signals in the spleen and whole blood further underscores the importance of systemic immune activation in IBD [4]. Using the gsMap algorithm to map GWAS signals onto the spatial transcriptomic atlas of E16.5 mouse embryos, we observed common spatial enrichment of PSC and UC genetic signals in the gastrointestinal tract. This finding provides spatial-dimensional genetic support for the “gut–liver axis” hypothesis from a developmental biology perspective, suggesting that the comorbid relationship between PSC and UC may be rooted in shared gene expression regulatory programs during digestive system development.

### 4.2. NK Cells: The Core Immune Effector Cell Type in PSC–UC Comorbidity

A notable finding of this study is the identification of NK cells as the core effector cell type underlying PSC–UC comorbidity through multi-dimensional evidence integration. It is important to formally address why seismicGWAS and ECLIPSER did not independently identify NK cells as a statistically significant cell type, despite their prioritization by CELLECT and single-cell differential analysis. Several methodological factors likely contribute to this discrepancy. First, seismicGWAS computes a cell-type specificity score that rewards genes expressed at consistently higher levels across all cells within a given cell type; NK cells, which represent a relatively rare population in the datasets analyzed, may exhibit higher transcriptional heterogeneity, thereby reducing the specificity score despite their biological relevance. Second, ECLIPSER relies on the enrichment of GWAS risk loci within cell-type-specific differentially expressed genes identified from the single-cell atlases used in this study; given the modest sample sizes of the PSC and UC single-cell datasets, the statistical power to identify cell-type-specific differential expression patterns may be insufficient to drive ECLIPSER enrichment to significance. Third, the CELLECT framework employs the Tabula Muris reference, which encompasses a broader range of cell types and may better capture the heritability contribution of NK cells through its gene set-based heritability enrichment approach, which is less sensitive to single-cell dataset sample size. Taken together, the failure of seismicGWAS and ECLIPSER to reach significance should be interpreted in the context of method-specific sensitivity, dataset-specific limitations, and the inherent challenge of detecting enrichment for rare cell types in small single-cell cohorts.

NK cells are core effector cells of the innate immune system, and recent studies have amply demonstrated their functional diversity across different tissues [29]. NK cells exhibit remarkable phenotypic and functional heterogeneity in different tissue microenvironments, with their tissue-resident properties and local immunoregulatory functions far exceeding classical understanding [29,30]. In the liver, NK cells account for a substantial proportion of hepatic lymphocytes and participate in diverse functions including anti-infection defense, immune surveillance, and immunoregulation [29]. In the pathological context of PSC, activated NK cells can secrete pro-inflammatory cytokines such as interferon-γ (IFN-γ) and tumor necrosis factor-α (TNF-α), directly injuring biliary epithelial cells or promoting periductal inflammatory reactions [1]. In the intestine, dysfunction of NK cells and innate lymphoid cells has been established to be closely linked to the pathogenesis of IBD [4,30].

Notably, our findings position NK cells at the intersection of the PSC–UC “gut–liver axis.” According to the gut–liver axis hypothesis, gut-derived immune cells can migrate to the liver via the portal venous circulation [5]. As innate immune cells with high migratory capacity, NK cells may serve as the critical “messenger” cells that relay intestinal inflammatory signals to the liver. NK cells activated within the intestinal inflammatory microenvironment may enter the liver through the portal vein and exert pro-inflammatory and pro-fibrotic effects in the periductal region, thereby establishing a cellular-level link between the intestinal inflammation of UC and the bile duct injury of PSC. This hypothesis provides a novel immune cell-based mechanistic framework for explaining the high comorbidity rate between PSC and UC.

### 4.3. STAT3: The Hub Gene Linking the Immunopathological Mechanisms of PSC and UC

Through cross-validation by five independent analytical algorithms—eCAVIAR colocalization, fastENLOC colocalization, Open4Gene chromatin accessibility analysis, hdWGCNA co-expression network analysis, and scPrediXcan cell-type-specific TWAS—*STAT3* was identified as the sole high-confidence key gene. *STAT3* (signal transducer and activator of transcription 3) is a central effector molecule of the JAK–STAT signaling pathway [31] and plays an indispensable role in immune cell differentiation, activation, survival, and inflammatory response regulation [32]. In NK cell biology, *STAT3* is a key downstream mediator of cytokine signaling from interleukin-21 (IL-21) and interleukin-15 (IL-15), among others, regulating NK cell maturation, activation, and cytotoxic function [31,32].

In the pathological context of PSC, sustained *STAT3* activation can drive biliary epithelial cell proliferation and inflammatory mediator secretion, further recruiting immune cells to the periductal region and establishing a self-perpetuating inflammatory positive-feedback loop [1]. In UC, hyperactivation of the IL-6/*STAT3* signaling pathway promotes Th17 cell differentiation, suppresses regulatory T cell function, and drives the chronicity of intestinal mucosal inflammation [4,33]. Thus, *STAT3* as a shared key gene for PSC and UC may simultaneously drive hepatic and intestinal immunopathological processes through aberrant activation in NK cells and other immune cells, providing a molecular-level explanation for the comorbid relationship between the two diseases.

Exon-level analysis further revealed the potential association between tissue-differential expression of the *STAT3* gene and alternative splicing events. While isoform-specific quantification was not performed in the present study, the exon-level expression heterogeneity across tissues raises the intriguing hypothesis that tissue-specific splicing regulation may contribute to the differential functional effects of *STAT3* in the liver versus the colon. This possibility merits formal investigation using sQTL-based isoform analyses in future work. *STAT3* has at least two major splice isoforms—*STAT3α* and *STAT3β*—which differ significantly in function [34,35,36]. *STAT3α* possesses a complete transactivation domain and primarily exerts pro-inflammatory and pro-survival effects, whereas *STAT3β*, truncated at the C-terminus, displays certain inhibitory functions. The alternative splicing events observed in the Exon 1–4, 5–7, 14–19, and 21–25 regions suggest that tissue-specific splicing patterns of *STAT3* may determine its differential functional effects in the liver and colon. The high expression of *STAT3* in EBV-transformed lymphocytes is consistent with the MAGMA tissue enrichment results, further supporting the central role of *STAT3* in immune cell function.

### 4.4. Genetic Architecture Complexity of the STAT3 Locus

Colocalization analysis identified the high-risk variant rs3736161 within the *STAT3* locus on chromosome 17. This SNP is located directly within the *STAT3* gene region, suggesting that this genetic variant may influence PSC and UC disease risk by directly regulating *STAT3* expression or splicing patterns. More importantly, GCTA-COJO conditional analysis revealed 35 independent association signals within this region in addition to the lead SNP rs3736161 (including rs1053004). This allelic heterogeneity indicates that the *STAT3* locus is regulated by multiple independent functional variants, which may independently contribute to disease risk through different molecular mechanisms [6]. The clinical implication is that therapeutic strategies targeting the *STAT3* pathway may need to account for individual-level variability across different genetic backgrounds.

### 4.5. Clinical Translational Implications

Our findings offer several insights for the clinical management of PSC–UC comorbidity. First, the identification of NK cells as core effector cells suggests that monitoring the number and functional status of NK cells in peripheral blood or tissue may aid in assessing disease activity and progression risk in patients with PSC–UC comorbidity. Second, the identification of *STAT3* as a key hub gene provides a theoretical basis for targeted therapeutic strategies. Multiple inhibitors targeting *STAT3* and the JAK–STAT pathway (including ruxolitinib, tofacitinib, filgotinib, and upadacitinib) have entered clinical use or trial stages [32,33,34]. Notably, JAK inhibitors such as upadacitinib have demonstrated significant clinical benefits in the treatment of UC [37], and their efficacy may be partly mediated through the suppression of excessive *STAT3* activation in NK cells. This hypothesis warrants further investigation in clinical studies of PSC–UC comorbidity patients and provides a theoretical rationale for exploring the potential therapeutic value of JAK inhibitors in PSC.

### 4.6. Limitations

Several limitations of this study should be addressed in future work. First, the GWAS data were derived primarily from European-ancestry cohorts, and the generalizability of the findings to other ethnic populations requires further validation. Second, the relatively limited sample size of the single-cell transcriptomic data may have affected the statistical power of certain analyses; larger single-cell cohorts are needed for validation. Third, this study was primarily based on computational biology analyses; although cross-validation by multiple algorithms enhanced the reliability of the results, the specific molecular mechanisms by which *STAT3* mediates PSC–UC comorbidity in NK cells require verification through in vitro functional experiments and animal models. Fourth, the single-cell multiome data used for Open4Gene analysis were derived from healthy donor PBMCs and may not directly reflect disease-state chromatin accessibility changes. Fifth, the comorbid relationship between PSC and UC may also involve other dimensions such as the gut microbiome and metabolome, which were not addressed in this study. Additionally, no formal genome-wide genetic correlation analysis between PSC and UC was performed in the present study; incorporating such cross-trait analyses in future work would provide a direct quantitative estimate of shared genetic architecture to complement the multi-omics integrative findings reported here. Future research should integrate multi-dimensional omics data, validate the present findings in larger multi-center, multi-ethnic cohorts, and elucidate the precise molecular mechanisms of the *STAT3*/NK cell axis in gut–liver immune crosstalk through functional experiments.

## 5. Conclusions

By constructing a multi-layered integrative analytical framework spanning GWAS to single-cell transcriptomics, this study systematically uncovered the shared immunogenetic mechanisms underlying PSC–UC comorbidity. The results demonstrate that (1) the intestine and immune-related tissues are key tissues commonly enriched for the genetic signals of PSC and UC, supporting the important role of the “gut–liver axis” in comorbidity pathogenesis; (2) through multi-dimensional evidence integration across four complementary methods, NK cells were consistently identified as the core immune effector cell type in PSC–UC comorbidity, revealing the pivotal role of the innate immune system in gut–liver immune crosstalk; (3) *STAT3* was cross-validated by five independent algorithms as the sole high-confidence key comorbidity gene, and its broad expression in NK cells and multiple immune cell types, together with its tissue-differential alternative splicing patterns, suggests that *STAT3* may simultaneously regulate hepatic and intestinal immunopathological processes through different molecular mechanisms; and (4) multiple independent functional variants, represented by rs3736161, exist within the *STAT3* locus, revealing a complex genetic regulatory architecture at this site. In summary, this study is the first to identify the NK cell–*STAT3* axis as the central molecular nexus linking the immunopathological mechanisms of PSC and UC from a multi-omics integrative perspective. These findings provide novel genetic evidence for understanding the gut–liver immune axis in comorbidity and lay a theoretical foundation for developing therapeutic strategies targeting the *STAT3*/NK cell axis.

## Figures and Tables

**Table 1:** Cell type enrichment of e/sQTL-mapped genes for PSC and UC GWAS locus sets in liver and thyroid using ECLIPSER.

Trait	Tissue	Cell Type	Fold-Enrichment	Fold-Enrichment (95% CI)	*p*
PSC	Liver	B cell	1.046	0.695, 1.448	0.406
PSC	Liver	Hepatocytes	0.877	0.591, 1.197	0.779
PSC	Liver	Monocyte	1.109	0.844, 1.370	0.212
PSC	Liver	NK cell	1.204	0.925, 1.478	0.077
UC	Intestine	B cell	0.989	0.942, 1.000	0.975
UC	Intestine	Endothelial cells	0.989	0.943, 1.000	0.975
UC	Intestine	Epithelial cells	0.989	0.943, 1.000	0.975
UC	Intestine	Monocyte	0.989	0.942, 1.000	0.975
UC	Intestine	NK cell	0.989	0.943, 1.000	0.975
UC	Intestine	Neurons	0.989	0.943, 1.000	0.975
UC	Intestine	Smooth muscle cells	0.989	0.943, 1.000	0.975
UC	Intestine	T cells	0.989	0.943, 1.000	0.975

PSC, Primary Sclerosing Cholangitis; UC, Ulcerative Colitis; CI, Confidence Interval.

**Table 2:** Cell type prioritization in PSC and UC.

Cell Type	Trait	Cell Atlas	seismicGWAS	ECLIPSER	CELLECT	Total
B cell	PSC	0	0	0	0	0
Hepatocytes	PSC	1	0	0	0	1
Monocyte	PSC	0	0	0	0	0
NK cell	PSC	1	0	0	2	3
B cell	UC	0	0	0	0	0
Endothelial cells	UC	0	0	0	0	0
Epithelial cells	UC	0	0	0	0	0
Monocyte	UC	0	0	0	0	0
NK cell	UC	0	0	0	2	2
Neurons	UC	0	0	0	0	0
Smooth muscle cells	UC	0	0	0	0	0
T cells	UC	0	0	0	0	0

PSC, Primary Sclerosing Cholangitis; UC, Ulcerative Colitis; seismicGWAS and ECLIPSER failed to provide prioritization results.

**Table 3:** Evidence Thresholds Across Five Gene Prioritization Methods.

Method	Level of Evidence: High	Level of Evidence: Medium	Level of Evidence: Low
hdWGCNA	YES	YES/NO	YES/NO
eCAVIAR	CLPP ≥ 0.8	CLPP ≥ 0.7	CLPP ≥ 0.01
fastENLOC	RCP ≥ 0.9	RCP ≥ 0.7	RCP ≥ 0.1
Open4Gene	YES	YES/NO	YES/NO
scPrediXcan	YES	YES/NO	YES/NO

CLPP, colocalization posterior probability; RCP, regional colocalization probability; “YES” indicates that the gene was identified as a hub gene within the M1 co-expression module in NK cells; for Open4Gene, “YES” indicates the presence of a significant cis-peak–gene regulatory pair (hurdle.Res.zero.*p* > 0.05, hurdle.Res.count.*p* ≤ 0.05 and spearman.*p* ≤ 0.05); and for scPrediXcan, “YES” indicates a statistically significant gene–trait association in NK cells (*p* < 0.05).

## Data Availability

The datasets analyzed during the current study are available in the finngen database repository, https://www.finngen.fi/en (IDs: K11_CHOLANGI and K11_UC_STRICT2, accessed on 13 December 2025). The datasets analyzed in the current study are available in the IEU OpenGWAS repository, https://opengwas.io/ (IDs: ieu-a-1112 and ukb-b-19386, accessed on 16 December 2025). The datasets analyzed in the current study are available in the GEO repository, https://www.ncbi.nlm.nih.gov/geo/ (IDs: GSE247128 and GSE250487, accessed on 28 January 2026). The datasets analyzed during the current study are available in the 10× Genomics repository, https://www.10xgenomics.com/datasets/pbmc-from-a-healthy-donor-granulocytes-removed-through-cell-sorting-10-k-1-standard-1-0-0 (single-cell multi-omics data from healthy human PBMCs, accessed on 16 December 2025). The datasets analyzed in the current study are available in the GTEx repository, https://gtexportal.org/home/datasets (eQTL and sQTL summary statistics, accessed on 15 January 2026). The datasets analyzed in the current study are available in the MSigDB repository, http://www.gsea-msigdb.org/gsea/msigdb/collections.jsp, accessed on 10 December 2025 (Gene Ontology, Reactome, and KEGG gene sets). The datasets analyzed in the current study are available in the Mouse Genome Informatics (MGI) repository, http://www.informatics.jax.org/, accessed on 10 December 2025 (Mouse Phenotype Ontology gene sets).

## Supplementary Materials

The following supporting information can be downloaded at: https://www.mdpi.com/article/10.3390/life16060950/s1, Figure S1: Cluster dendrograms of single-cell transcriptome data at different resolutions for PSC. Figure S2: Cluster dendrograms of single-cell transcriptome data at different resolutions for UC. Table S1: QTLEnrich results for PSC and UC GWAS meta-analyses and e/sQTLs from 49 GTEx tissues. Table S2: Tissue enrichment of PSC and UC associations using MAGMA. Table S3: Gene set enrichment analysis of target genes of e/sQTLs using GeneEnrich. Table S4: Cell type enrichment of PSC and UC associations using S-LDSC and MAGMA. Table S5: Summary table of colocalizing e/sGenes and enriched for PSC and UC GWAS loci. Table S6: Results of Open Chromatin to Gene Expression Analysis. Table S7: Summary Results of scPrediXcan Analysis. Table S8: Conditional analysis of risk loci within the rs3736161 locus.

## Abbreviations

The following abbreviations are used in this manuscript:

CELLECT	Cell-type Expression-specific Integration for Complex Traits
CLPP	Colocalization posterior probability
COJO	Conditional and joint analysis
ECLIPSER	Enrichment of Cell-type-specific Loci Identified by Phenotypic SNP Enrichment with Regulatory annotations
eQTL	Expression quantitative trait locus
FUMA	Functional Mapping and Annotation
GCTA	Genome-wide Complex Trait Analysis
GEO	Gene Expression Omnibus
GO	Gene Ontology
gsMap	Genetically informed spatial mapping of cells for complex traits
GTEx	Genotype-Tissue Expression (project)
GWAS	Genome-wide association study
hdWGCNA	High-dimensional weighted gene co-expression network analysis
HLA	Human leukocyte antigen
JAK	Janus kinase
KEGG	Kyoto Encyclopedia of Genes and Genomes
LD	Linkage disequilibrium
MAF	Minor allele frequency
MAGMA	Multi-marker Analysis of GenoMic Annotation
MGI	Mouse Genome Informatics
MHC	Major histocompatibility complex
MLP	Multi-layer perceptron
MSigDB	Molecular Signatures Database
NK cell	Natural killer cell
PBMC	Peripheral blood mononuclear cell
PCA	Principal component analysis
PSC	Primary sclerosing cholangitis
QC	Quality control
RCP	Regional colocalization probability
scATAC-seq	Single-cell assay for transposase-accessible chromatin sequencing
scRNA-seq	Single-cell RNA sequencing
sc-ST	Single-cell spatial transcriptomics
S-LDSC	Stratified linkage disequilibrium score regression
SNP	Single nucleotide polymorphism
sQTL	Splicing quantitative trait locus
STAT3	Signal transducer and activator of transcription 3
Th17	T helper 17 (cell)
TNF-α	Tumor necrosis factor-alpha
TOM	Topological overlap matrix
Treg	Regulatory T cell
TSS	Transcription start site
TWAS	Transcriptome-wide association study
UC	Ulcerative colitis
UMAP	Uniform Manifold Approximation and Projection
UMI	Unique molecular identifier

## References

[1] Karlsen T.H., Folseraas T., Thorburn D., Vesterhus M. (2017). Primary Sclerosing Cholangitis—A Comprehensive Review. J. Hepatol..

[2] Kobayashi T., Siegmund B., Le Berre C., Wei S.C., Ferrante M., Shen B., Bernstein C.N., Danese S., Peyrin-Biroulet L., Hibi T. (2020). Ulcerative Colitis. Nat. Rev. Dis. Prim..

[3] Awoniyi M., El Hag M., Hernandez J., Yang Q., Evans N., Nemet I., Ngo B., Coskuner D., Zhou J., Farmer M. (2026). Dysbiotic Microbiota Trigger Colitis-Associated Colorectal Cancer and Imprint a Distinctive Bile Acid Profile in a PSC-IBD Model. Gut.

[4] Friedrich M., Pohin M., Powrie F. (2019). Cytokine Networks in the Pathophysiology of Inflammatory Bowel Disease. Immunity.

[5] Albillos A., de Gottardi A., Rescigno M. (2020). The Gut-Liver Axis in Liver Disease: Pathophysiological Basis for Therapy. J. Hepatol..

[6] Ji S.-G., Juran B.D., Mucha S., Folseraas T., Jostins L., Melum E., Kumasaka N., Atkinson E.J., Schlicht E.M., Liu J.Z. (2017). Genome-Wide Association Study of Primary Sclerosing Cholangitis Identifies New Risk Loci and Quantifies the Genetic Relationship with Inflammatory Bowel Disease. Nat. Genet..

[7] Ellinghaus D., Jostins L., Spain S.L., Cortes A., Bethune J., Han B., Park Y.R., Raychaudhuri S., Pouget J.G., Hübenthal M. (2016). Analysis of Five Chronic Inflammatory Diseases Identifies 27 New Associations and Highlights Disease-Specific Patterns at Shared Loci. Nat. Genet..

[8] GTEx Consortium (2020). The GTEx Consortium Atlas of Genetic Regulatory Effects across Human Tissues. Science.

[9] Gamazon E.R., Wheeler H.E., Shah K.P., Mozaffari S.V., Aquino-Michaels K., Carroll R.J., Eyler A.E., Denny J.C., Nicolae D.L., GTEx Consortium (2015). A Gene-Based Association Method for Mapping Traits Using Reference Transcriptome Data. Nat. Genet..

[10] Timshel P.N., Thompson J.J., Pers T.H. (2020). Genetic Mapping of Etiologic Brain Cell Types for Obesity. eLife.

[11] Watanabe K., Umićević Mirkov M., de Leeuw C.A., van den Heuvel M.P., Posthuma D. (2019). Genetic Mapping of Cell Type Specificity for Complex Traits. Nat. Commun..

[12] Song L., Chen W., Hou J., Guo M., Yang J. (2025). Spatially Resolved Mapping of Cells Associated with Human Complex Traits. Nature.

[13] Hao Y., Stuart T., Kowalski M.H., Choudhary S., Hoffman P., Hartman A., Srivastava A., Molla G., Madad S., Fernandez-Granda C. (2024). Dictionary Learning for Integrative, Multimodal and Scalable Single-Cell Analysis. Nat. Biotechnol..

[14] Korsunsky I., Millard N., Fan J., Slowikowski K., Zhang F., Wei K., Baglaenko Y., Brenner M., Loh P., Raychaudhuri S. (2019). Fast, Sensitive and Accurate Integration of Single-Cell Data with Harmony. Nat. Methods.

[15] Aran D., Looney A.P., Liu L., Wu E., Fong V., Hsu A., Chak S., Naikawadi R.P., Wolters P.J., Abate A.R. (2019). Reference-Based Analysis of Lung Single-Cell Sequencing Reveals a Transitional Profibrotic Macrophage. Nat. Immunol..

[16] Lai Q., Dannenfelser R., Roussarie J.-P., Yao V. (2025). Disentangling Associations between Complex Traits and Cell Types with Seismic. Nat. Commun..

[17] Eraslan G., Drokhlyansky E., Anand S., Fiskin E., Subramanian A., Slyper M., Wang J., Van Wittenberghe N., Rouhana J.M., Waldman J. (2022). Single-Nucleus Cross-Tissue Molecular Reference Maps toward Understanding Disease Gene Function. Science.

[18] Rouhana J.M., Wang J., Eraslan G., Anand S., Hamel A.R., Cole B., Regev A., Aguet F., Ardlie K.G., Segrè A.V. (2021). ECLIPSER: Identifying Causal Cell Types and Genes for Complex Traits through Single Cell Enrichment of e/sQTL-Mapped Genes in GWAS Loci. bioRxiv.

[19] Finucane H.K., Reshef Y.A., Anttila V., Slowikowski K., Gusev A., Byrnes A., Gazal S., Loh P.-R., Lareau C., Shoresh N. (2018). Heritability Enrichment of Specifically Expressed Genes Identifies Disease-Relevant Tissues and Cell Types. Nat. Genet..

[20] Schaum N., Karkanias J., Neff N.F., May A.P., Quake S.R., Wyss-Coray T., Darmanis S., Batson J., Botvinnik O., Chen M.B. (2018). Single-Cell Transcriptomics of 20 Mouse Organs Creates a Tabula Muris. Nature.

[21] Li Y., Dang X., Chen R., Teng Z., Wang J., Li S., Yue Y., Mitchell B.L., Zeng Y., Yao Y.-G. (2025). Cross-Ancestry Genome-Wide Association Study and Systems-Level Integrative Analyses Implicate New Risk Genes and Therapeutic Targets for Depression. Nat. Hum. Behav..

[22] Morabito S., Reese F., Rahimzadeh N., Miyoshi E., Swarup V. (2023). hdWGCNA Identifies Co-Expression Networks in High-Dimensional Transcriptomics Data. Cell Rep. Methods.

[23] Hormozdiari F., van de Bunt M., Segrè A.V., Li X., Joo J.W.J., Bilow M., Sul J.H., Sankararaman S., Pasaniuc B., Eskin E. (2016). Colocalization of GWAS and eQTL Signals Detects Target Genes. Am. J. Hum. Genet..

[24] Wen X., Pique-Regi R., Luca F. (2017). Integrating Molecular QTL Data into Genome-Wide Genetic Association Analysis: Probabilistic Assessment of Enrichment and Colocalization. PLoS Genet..

[25] Liu H., Abedini A., Ha E., Ma Z., Sheng X., Dumoulin B., Qiu C., Aranyi T., Li S., Dittrich N. (2025). Kidney Multiome-Based Genetic Scorecard Reveals Convergent Coding and Regulatory Variants. Science.

[26] Zhou Y., Adeluwa T., Zhu L., Salazar-Magaña S., Sumner S., Kim H., Gona S., Nyasimi F., Kulkarni R., Powell J.E. (2025). scPrediXcan Integrates Deep Learning Methods and Single-Cell Data into a Cell-Type-Specific Transcriptome-Wide Association Study Framework. Cell Genom..

[27] Yang J., Lee S.H., Goddard M.E., Visscher P.M. (2011). GCTA: A Tool for Genome-Wide Complex Trait Analysis. Am. J. Hum. Genet..

[28] Pruim R.J., Welch R.P., Sanna S., Teslovich T.M., Chines P.S., Gliedt T.P., Boehnke M., Abecasis G.R., Willer C.J. (2010). LocusZoom: Regional Visualization of Genome-Wide Association Scan Results. Bioinformatics.

[29] Dogra P., Rancan C., Ma W., Toth M., Senda T., Carpenter D.J., Kubota M., Matsumoto R., Thapa P., Szabo P.A. (2020). Tissue Determinants of Human NK Cell Development, Function, and Residence. Cell.

[30] Vivier E., Artis D., Colonna M., Diefenbach A., Di Santo J.P., Eberl G., Koyasu S., Locksley R.M., McKenzie A.N.J., Mebius R.E. (2018). Innate Lymphoid Cells: 10 Years On. Cell.

[31] Hu X., Li J., Fu M., Zhao X., Wang W. (2021). The JAK/STAT Signaling Pathway: From Bench to Clinic. Signal Transduct. Target. Ther..

[32] Zou S., Tong Q., Liu B., Huang W., Tian Y., Fu X. (2020). Targeting STAT3 in Cancer Immunotherapy. Mol. Cancer.

[33] Johnson D.E., O’Keefe R.A., Grandis J.R. (2018). Targeting the IL-6/JAK/STAT3 Signalling Axis in Cancer. Nat. Rev. Clin. Oncol..

[34] Weiss S., Zdársky B., Witalisz-Siepracka A., Edtmayer S., Holzer A., Heindl K., Casanova E., Podar K., Stoiber D. (2025). Atovaquone and Selinexor as a Novel Combination Treatment Option in Acute Myeloid Leukemia. Cancer Lett..

[35] Kise M., Masaki S., Kataoka N., Suzuki K. (2024). RNA Binding Protein CUGBP2/ETR-3 Regulates STAT3 Alternative Splicing. Biochem. Biophys. Res. Commun..

[36] Cheng G., Sui C., Xu Y., Lu W., Li X. (2025). RNA Splicing of the STAT3 by PCBP1 Promotes Vulnerable Plaque Formation via Macrophage-like Phenotype Modulation of Vascular Smooth Muscle Cell. Atherosclerosis.

[37] Danese S., Vermeire S., Zhou W., Pangan A.L., Siffledeen J., Greenbloom S., Hébuterne X., D’Haens G., Nakase H., Panés J. (2022). Upadacitinib as Induction and Maintenance Therapy for Moderately to Severely Active Ulcerative Colitis: Results from Three Phase 3, Multicentre, Double-Blind, Randomised Trials. Lancet.

